# Early experiences of radiographers in Ireland during the COVID-19 crisis

**DOI:** 10.1186/s13244-020-00910-6

**Published:** 2020-09-25

**Authors:** Shane J. Foley, Anne O’Loughlin, Jill Creedon

**Affiliations:** 1 Radiography & Diagnostic Imaging, School of Medicine, University College Dublin, Dublin 4, Ireland; 2 Department of Radiology, Cork University Hospital, Cork, Ireland

**Keywords:** Coronavirus, COVID-19, Radiographers, Infection control, Burnout

## Abstract

**Background:**

Imaging is crucial for assessing the severity and progression of COVID-19. Radiographers are amongst the first-line health professionals that may be exposed to infected persons. This work describes the early experience of radiographers in Ireland to the impact of COVID-19 using two electronic surveys distributed 6 weeks apart. Results were analysed using descriptive statistics and thematic analysis.

**Results:**

A total of 370 responded to the first survey and 276 the second, with all six Irish health regions represented. Three quarters of radiographers (77%) reported having adequate personal protective equipment (PPE) available to them. However, almost half of the radiographers were inadvertently exposed to COVID-19-positive patients without appropriate PPE, largely attributed to poor communication and testing. Anxiety levels while initially high, reduced substantially 6 weeks into the crisis period. However, obvious distress was noted amongst some respondents. Forty percent of radiographers reported burnout symptoms due to the COVID-19 crisis and 30% reported considering changing jobs or retiring since the COVID-19 outbreak.

**Conclusion:**

Clear communication regarding changing protocols and importantly patients’ infectious status are essential to safeguard healthcare workers and to minimise unnecessary anxiety and distress. Attention is required to staff mental health including the identification of burnout symptoms to prevent long-term negative consequences of the pandemic on radiography services.

## Key points

- Up to 16% of radiographers in Ireland responded to an electronic survey.
- Almost half of the radiographers were inadvertently exposed to COVID-19-positive patients without appropriate PPE.
- Anxiety levels reduced substantially 6 weeks into the crisis period.
- Forty percent of radiographers in Ireland reported burnout symptoms due to the COVID-19 crisis.
- Thirty percent reported considering changing jobs or retiring since the COVID-19 outbreak.

## Background

The global pandemic of the novel coronavirus, COVID-19, has spread rapidly worldwide, with countries and health services adapting to limit the potential spread and to manage the consequences of the virus on populations. Governments have instituted restrictions on movement and gatherings of people, while health services have had to rapidly organise mass testing and contact tracing capabilities, while also procuring extraordinary volumes of personal protective equipment (PPE). At a local level, hospitals and care facilities have had to swiftly change their practices to allow specific triaging of patients, increased infection control measures and alterations to work patterns (e.g. team working based on shift patterns) to safeguard both patients and staff.

The first confirmed case of COVID-19 in Ireland was presented on February 29 [1] with the first death occurring on March 11. This was followed by emergency measures being introduced by the government on March 24 and a stay-at-home order being issued on March 28 [2]. Ireland has one of the highest infection rates of COVID-19 among healthcare workers at 32% at the start of June 2020 [2] despite increased access to PPE and successful suppression of the virus in the community. Radiographers are among the first-line health professionals that may be exposed to infected persons. Although not required for diagnosis, imaging (typically chest X-ray or chest CT) is essential for assessing the severity and disease progression of COVID-19 [3]. Radiographers are therefore in frequent contact with confirmed positive patients but also suspected infected patients presenting with respiratory symptoms. Naturally, direct patient contact is required for such imaging studies, thus increasing their potential exposure to the virus.

In this paper, we describe the early experience of radiographers in Ireland to the occupational and psychological impact of the COVID-19 outbreak by way of two electronic surveys, one issued in late March and one in early May of 2020.

## Methods

The research design was based around the use of a two-stage questionnaire focussed on collating radiographers’ experience and perspective of working conditions during the initial Irish COVID-19 response (March 2020) with their response during the ending of initial emergency measures (late May 2020). This time period was not predetermined and was chosen based on the progress of COVID-19 in Ireland. An electronic questionnaire was selected to maximise the participation of respondents countrywide. Recruitment was voluntary and the survey link was posted on social media (Facebook and Twitter) specifically targeting Irish-based radiographers and also emailed to radiography service managers to distribute.

A questionnaire was developed containing mostly closed questions. Questions were broadly divided into four categories; demographics, infection control, occupational, and psychological impact. The questionnaire was first piloted on five radiographers to test for reliability and validity [4] with respondents asked to feedback specifically on question clarity, potential ambiguities in questions, missing or redundant questions, and any issues with flow/ordering. The survey was further edited before being distributed. As all responses were anonymous an exemption from the ethical review was obtained from the local academic institution (LS-E-20-57-Foley).

The first questionnaire contained 28 questions while the second a maximum of 35, although inbuilt logic resulted in respondents asked to complete a minimum of 23 and 24 questions respectively for both surveys. Both surveys contained eight common questions to allow a comparison of responses over time as the COVID situation evolved. Both surveys were left open for 2-week periods.

Responses were analysed using descriptive statistics for quantitative questions and thematic analysis of open response questions. Chi-squared tests were used to compare reported anxiety levels across the HSE regions and hospital sizes, using SPSS 24 (IBM Corp, USA).

## Results

A total of 370 responses were received for the first survey, and 266 for the second, corresponding to 16% and 11% of radiographers in Ireland (*n* = 2387) [5]. Responses were received from all six Irish healthcare regions (range: 5–27%) with as expected a predominance in the Dublin regions (Survey 1: 53%, Survey 2: 48%). In the initial survey, a majority (53%) reported a reduction in clinical workload with respondents citing the cancellation of outpatient clinics and non-essential work. However, 36% reported an initial increase in workload, this being attributed to the increasing volume of portable X-rays, out-of-hours work and time spent on infection control measures. Six weeks later, most respondents reported an increase in both daytime and out-of-hours workloads (46% and 62%, respectively) as non-essential work returned and non-COVID-19-related attendances increased. Many respondents commented that while imaging volumes had decreased, the time required per examination had increased substantially due to infection control requirements.

In March, just 33% of radiographers reported being appropriately prepared for new practices, protocols and procedures and when asked 6 weeks later 56% of respondents reported difficulty keeping up to date with protocol adjustments in the workplace. Initially, 71% felt supported by their employers via communication, workload sharing and psychological supports, which rose to 78% on the second survey as the number of respondents who indicated their employers were ‘not supportive at all’ reduced from 12% in March to 6% in May. There were many positive comments, all citing good communication and a sense of being cared for by management. Criticism of management initially mostly cited a lack of communication, delays in implementing safe working practices (in particular pod-based systems of work) or not being informed following exposure to COVID-19 positive patients and staff. Some radiographers specifically commented on the lack of social distancing between staff and a fear of ‘infecting each other’ (A55).

**Fig. 1:**
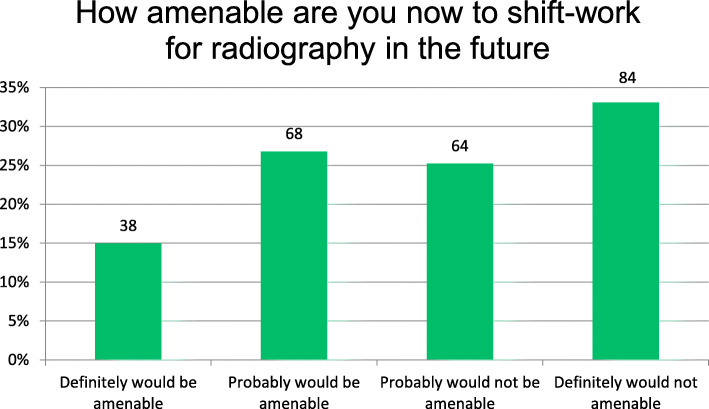
Percentage of respondents amenable to shift-work for radiography in the future (survey 2)

Twenty-seven percent reported being asked to perform roles typically outside of their job routine during the COVID-19 crisis, with most answering that conventional nursing tasks such as blood pressure and temperature checks, detaching and attaching IV fluids and feeding and cleaning soiled patients, were shared to avoid excessive use of PPE and the additional time required to don/doff. Others detailed performing more administrative roles—one person performing audits at home to accrue hours as they struggle with childcare. One radiographer even reported anointing a patient on behalf of a priest who could not enter an isolation room. Just 10% of respondents allowed other persons to carry out radiographer duties, with those who did in particular citing radiography students and radiography aides being delegated more tasks. In the second survey, the cohort was split when asked how amenable they would be to shift-work in radiography in the future with 58% saying they would not be amendable, most of this cohort stating they would ‘definitely not be amenable’ (Fig. 1). Many strong comments were made against shift-work which referred to either quality of life issues, lack of adequate staffing, or to potential poorer financial remuneration. In the subsequent open comment field, approximately one third of comments supplied referred to discontent with current salary levels. Those who were positive about shift-work prefaced this with comments detailing the need for additional staff resources and fair remuneration.

### Infection control

**Fig. 2:**
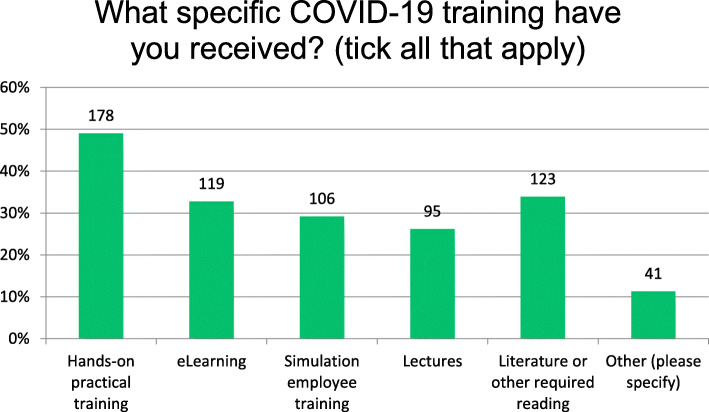
What specific COVID-19 training respondents received (survey 1)

While 20% of respondents in the initial survey had not had any exposure to COVID-19 patients, 6 weeks later, 97% of respondents reported COVID-19 patients attending their centres. When initially surveyed, 92% reported having a thorough understanding of infection control and prevention of COVID-19, yet 26% felt they were not appropriately trained for new infection control procedures at the outset of the pandemic. While most respondents reported receiving a range of training (Fig. 2), a small proportion reported receiving no training at all (*n* = 18).

Although 93% expressed confidence that their infection control practices are safe and have no fear of infecting patients, 75% were concerned for the safety of household members due to their own exposure. A clear majority (87%) reported taking extra measures on returning home to reduce the risk of COVID-19 spread. Of these responses, most referred to showering on returning home (69%) and washing clothes (47%). Thirty-two radiographers (17%) referred to cleaning surfaces while ten respondents (5%) referred to cleaning their phones. Just five respondents (2%) applied for temporary accommodation for healthcare workers as facilitated by the government, with four being successful. However, others commented on how due to living with vulnerable persons they had arranged their own alternative accommodation (*n* = 2), altered home sleeping arrangements (*n* = 6) or enforced social distancing at home (*n* = 8), while five others reported that family members had moved house to avoid potential exposure.

**Fig. 3:**
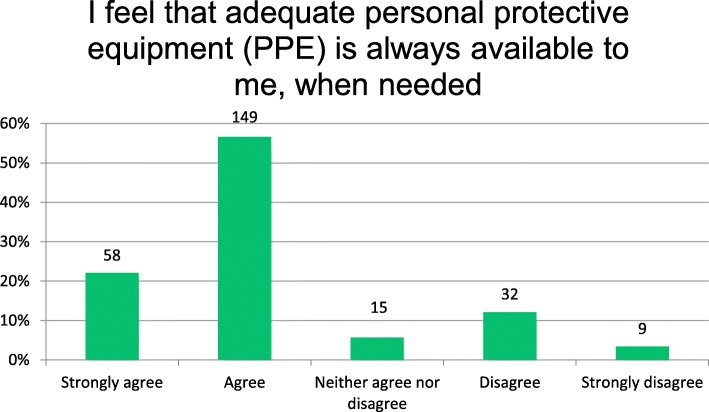
Availability of personal protective equipment (PPE) (survey 2)

Most radiographers (77%) reported having adequate PPE available to them with 16% disagreeing and reporting that they felt pressurised to engage with suspected/positive COVID-19 patients without adequate PPE (Fig. 3). However, many comments echoed anxiety about access to PPE as it was being ‘prioritised for [COVID] ward workers…and we are not being advised to wear the same amount of PPE’ (A15) and confusion about the appropriate use of the same, as ‘the rules on PPE keep changing’ (A83).

In the first survey, 23% reported having imaged a patient who was not initially suspected of having COVID-19 but later tested positive (although the majority 47% did not know or were unsure), and of these, 60% (*n* = 49) had not taken appropriate infection control measures at the time. Six weeks later, 45% of radiographers reported having had an inadvertent exposure to COVID-19-positive patients without appropriate PPE with a further 26% unsure. Most of these radiographers (83%, *n* = 44) went on to have direct contact with many more patients before realising the earlier patient was positive, with 21 radiographers reporting contact with over ten patients each. One comment was particularly enlightening

‘I’ve tested positive today after minimum exposure. I was sick early last week and refused testing 3 times by the [Health Service Executive] HSE. But eventually tested last night & [was] positive. In the meantime, I have exposed hundreds of people’. (A181)

Ninety percent of the exposed radiographers would have liked to have been tested for COVID-19 following this exposure although only 8% were. Just 29% are being regularly screened (via temperature testing) in their workplaces, with 68% stating they would be more at ease if this was being conducted. Many radiographers expressed a dissatisfaction with the approach to testing staff.

### Psychological impact/anxiety

Participants were asked to rate how anxious they feel regarding the COVID-19 virus in both surveys. Initially, more than 97% reported some level of anxiety with 77% reporting being moderately to extremely anxious in work and 71% while at home or in the community, but 6 weeks later, there was far less concern with 23% at home, 50% in the community, and 58% in work reporting similar levels of anxiety. Reported extreme anxiety in work reduced from 22 to 5% in the period. No statistically different anxiety levels were noted between either the HSE regions or hospital sizes when tested using chi-squared tests (*p* > 0.05). Some notable examples of distress were evident in both surveys, with references to stress and feeling vulnerable or overwhelmed.

‘I feel so vulnerable right now… I feel genuinely afraid for my life and the life of my family members at work right now’. (A99)

Although 70% of radiographers were aware of mental health resources available to them as frontline staff, just 10% have utilised such support. Comments related to anxiety reduced substantially in the second survey with even some examples of positive attitudes with respondents citing feeling ‘a bigger purpose in going to work (B24)’ or indeed becoming ‘more resilient’ (B184). Initially, a sizeable proportion of radiographers (63%, *n* = 213) reported experiencing social discomfort from family/friends because of their work in a centre with potential exposure to COVID-19, 22% reported their partner/family member/housemate had been negatively affected, including childcare, social distancing from relatives, some spouses being sent home from work. Several radiographers commented that their spouses’ workplace was concerned and that some spouses were given their own office or not allowed to use the staff canteen. Childcare issues were highlighted, with one radiographer saying their babysitter had quit due to the potential risk and another stating no-one would agree to mind their children for fear of infection. In the second survey, although childcare was not applicable to most respondents (58%), 28% reported difficulty in accessing childcare since the COVID-19 outbreak in Ireland.

When asked to rate whether radiographers are seen as an essential part of the health team in the response to COVID-19, initially 55% agreed although many comments were received about how radiographers were invisible both to colleagues internally, but also externally in particular by the media. Of the 89 comments provided on this question, many referenced feeling ‘forgotten’ ‘unappreciated’, ‘not recognised’ and ’omitted from decision making’. Others referred to a lack of ‘professional respect’ and communication towards radiographers. When later asked if radiographers felt more valued as a team member in a variety of clinical departments (intensive care, operating room, emergency department, wards), most radiographers felt no difference although twice as many felt more valued than less (23% vs 11%). Respondents were also asked to self-report any symptoms of burnout using a single-item response as per Knox et al. (2018) with 40% reporting symptoms to varying degrees (Table 1).

**Table 1:** Responses to whether radiographers had any symptoms of ‘burn-out’? (survey 2)

Due to the current COVID-19 crisis, do you have any of the following symptoms of ‘burnout’?		
	Responses	
I enjoy my work. I have no symptoms of burnout	9.5%	25
Occasionally I am under stress, and I don’t always have as much energy as I once did, but I don’t feel burned out	50.6%	133
I am definitely burning out and have one or more symptoms of burnout, such as physical and emotional exhaustion	29.3%	77
The symptoms of burnout that I am experiencing will not go away. I think about work frustrations a lot	8.4%	22
I feel completely burned out and often wonder if I can go on. I am at the point where I may need some changes or may need to seek some sort of help	2.3%	6

**Fig. 4:**
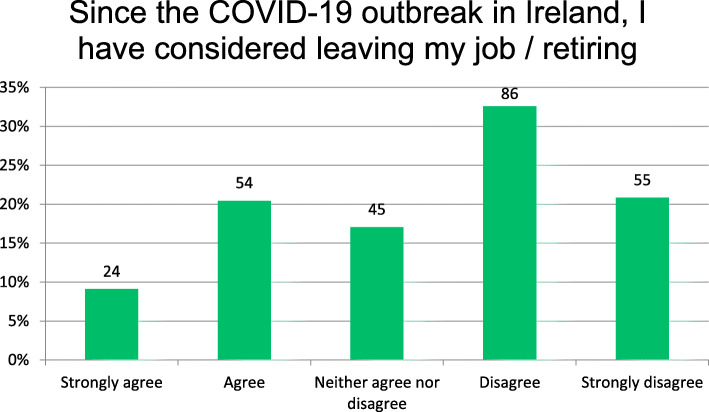
Since the COVID-19 outbreak in Ireland, I have considered leaving my job/retiring

Additionally, 30% reported considering changing jobs or retiring since the COVID-19 outbreak in Ireland (Fig. 4), with most comments referring to working conditions (40%) including workloads, exhaustion, feeling overwhelmed, stressed and undervalued. Childcare issues also featured (*n* = 7).

‘The hardest and darkest part of my career and I will work towards a career change when the economy recovers’. (B89)

## Discussion

This is the first study of radiographers in Ireland on the impact of the COVID-19 pandemic, and the large sample size is a representative sample from the Irish population with all regions and healthcare institutions being represented. The qualitative data provides rich contextual information and further insights to the quantitative results. Importantly, despite the worldwide shortages over personal protective equipment, radiographers in Ireland were largely satisfied that they had adequate supplies. This contrasts with a recent British Institute of Radiology study conducted in late April 2020, where two thirds of the 530 radiographer respondents reported inadequate levels of PPE [6]. The large difference in responses demonstrates variance in preparedness and supply between countries as demand for PPE escalated dramatically the world over with the epidemic. There was some variation noted here as 16% of respondents felt pressurised to engage with patients without adequate PPE. Guidance has been regularly evolving as new information on the virus becomes known, but this in itself was a considerable cause of anxiety amongst radiographers who felt they were not being adequately protected. This was evidenced by the high proportion of radiographers, 13% in the initial survey and 45% in the second, reporting having had an inadvertent exposure to COVID-19-positive patients without adequate PPE. This was largely attributed to poor communication—not being informed of patients (potential) infection status in advance of imaging. This is a cause for concern not just relative to increasing vectors of the disease but also heightened anxiety amongst radiographers which led to some feeling overwhelmed, an aspect supported by a recent Cochrane review [7]. Clear communication is essential not just to inform healthcare staff of new protocols but to ensure understanding and thus correct implementation.

Most radiographic examinations are quick and involve low patient contact activities [8], so the likely risk of disease transmission is low [9]. However, radiographers reported a high degree of anxiety relative to potentially cross-contaminating further patients or even their own families. Subsequent health service advice that all healthcare workers were to wear surgical masks when within 1 m of a person regardless of their COVID-19 status [8] will hopefully standardise practice and alleviate this anxiety further. There was also marked dissatisfaction as regards to testing of radiographers following such exposures, which further added to anxiety levels. Admittedly, the Irish response to instituting testing of the scale was slow and healthcare workers were not prioritised until late March for swab testing [10] after the first survey here was closed. National advice for temperature testing of employees was only introduced on May 8 [11], but the second survey revealed that less than one third of radiographers were being tested regularly and healthcare staff were asked to continue working until testing positive in many situations.

When asked what additional measures radiographers took to reduce the risk of spread, it was interesting to note that less than 4% followed full ECDC advice [12], which includes showering before leaving work where possible, having special shoes that are left in work, regularly cleaning and disinfecting electronic equipment such as mobile phones and cleaning the frequently touched surfaces in their car (e.g. steering wheel, knobs, screens). Many responses referred to a lack of facilities at work for showering, as noted elsewhere also [7] which is an important future consideration for hospital management to safeguard their employees but also public health. While only a small proportion of respondents indicated they cleaned their phones (5%) or common surfaces (*n* = 17%) as per ECDC guidelines, the survey question did ask for respondents to indicate additional measures they took related to COVID-19, so there is a possibility that many took these as routine steps. However, previously published work regarding mobile phone cleaning in radiography departments in Ireland showed quite a low general approach to such cleaning [13] and is worth reinforcing regularly as part of standard infection control updates and training. ECDC advice also recommends social distancing at home for healthcare professionals involved in managing COVID-19 patients which several respondents here referred to complying with, despite radiographers not actually managing such patients and thus not specifically included in this advice. Similarly, radiographers have put in place altered sleeping arrangements for family members at high risk which may not be required and may even be excessive. Clarifications and reminders to all healthcare staff would be useful to minimise excessive measures being followed unnecessarily, while also minimising the infection rates amongst staff.

Results here, in particular, the additional comments provided showed high levels of emotional stress and even distress among radiographers especially in the early stages of the COVID-19 pandemic. Healthcare worker responses to infectious disease pandemics are complicated and affected by feelings of vulnerability, loss of control and concern for the health of self and family [14] as evidenced here. Heightened anxiety levels have already been reported among front line workers during the COVID-19 pandemic with those involved in diagnosis more at risk to symptoms of anxiety, depression, insomnia and distress [15]. This resonates with the results and many of the comments provided here, which referred to poor communication, PPE concerns, a lack of social distancing and testing and quickly changing protocols rather than due to workload issues. It was interesting to note that a sizeable proportion of respondents encountered social discomfort as did their family members due to their potential exposure, which likely added to general anxiety levels. Despite a range of mental health resources being available to all healthcare workers, one third of respondents here were unaware of them and only 10% had utilised any. Managers and radiographer colleagues alike should be alert to mental health issues for colleagues, given the current lack of access to typical coping strategies such as social interactions and leisure activities. Worrying levels of distress were noted for some respondents here which, while less than the 50% reported following the SARS pandemic [16], highlights the importance of individual awareness and collegial support. Consideration should be given to regular promotion internally of mental health resources and staff meetings to inform but also debrief staff [17] while providing a useful forum for radiographers to share experiences and learnings.

Such events are especially important given the substantial amount of burnout reported in this work. High levels of stress are not uncommon in healthcare workers, but likely exacerbated in the wake of a global pandemic of a new infectious disease with information and control strategies continuously changing. Previous work reported high burnout rates of a small sample of radiographers in Ireland [18] and coupled with the considerable number of radiographer respondents in this work (30%) considering leaving their job or retiring since the COVID-19 outbreak, should be a concern for management and the profession alike. The potential exists for long-term negative effects on the current workforce [19] which may impact on services nationally unless stress and burnout among this profession are specifically addressed post pandemic. This will be especially important as the health service begins to address further demands due to increased backlogs as well as the potential for subsequent outbreaks.

### Limitations

Recruitment of respondents primarily via social media channels is likely to include recruitment bias due to the age profile typical of social media users, but this was offset by the distribution via email to radiography service managers. The slight reduction in response rate in the second survey likely reflects the aforementioned increasing clinical workloads and pressures coupled with the many surveys that started to circulate during the later period. Single-item questions were used to assess both anxiety and burnout in contrast to more detailed and validated questionnaires such as the Generalised Anxiety Disorder Scale or Maslach Burnout inventory. However, neither was the primary focus of the data collection here, and thus, single-item questions have a low response burden although they may substantially underestimate the actual level of burnout [20].

## Conclusion

Clear communication regarding changing protocols and importantly patients’ infectious status is essential to safeguard healthcare workers and to minimise unnecessary anxiety and distress. Attention is required to staff mental health including the identification of burnout symptoms to prevent long-term negative consequences of the pandemic on radiography services.

## Data Availability

The datasets used and/or analysed during the current study are available from the corresponding author on reasonable request

## Abbreviations

ECDC: European Centre for Disease Prevention and Control
FFP3: Filtering facepiece three
HSE: Health Service Executive
PPE: Personal protective equipment
